# REDbox: a comprehensive semantic framework for data collection and management in tuberculosis research

**DOI:** 10.1038/s41598-023-33492-6

**Published:** 2023-05-11

**Authors:** Vinícius Costa Lima, Rui Pedro Charters Lopes Rijo, Filipe Andrade Bernardi, Márcio Eloi Colombo Filho, Francisco Barbosa-Junior, Felipe Carvalho Pellison, Rafael Mello Galliez, Afrânio Lineu Kritski, Domingos Alves

**Affiliations:** 1 Ribeirão Preto Medical School, University of São Paulo, Ribeirão Preto, Brazil; 2 School of Technology and Management, Polytechnic Institute of Leiria, Leiria, Portugal; 3 Institute for Systems Engineering and Computers at Coimbra, Coimbra, Portugal; 4 Center for Research in Health Technologies and Services, Faculty of Medicine, University of Porto, Porto, Portugal; 5 Faculty of Medicine, Federal University of Rio de Janeiro, Rio de Janeiro, Brazil; 6 Department of Social Medicine, Ribeirão Preto Medical School, University of São Paulo, Ribeirão Preto, Brazil

**Keywords:** Software, Information technology, Computer science

## Abstract

Clinical research outcomes depend on the correct definition of the research protocol, the data collection strategy, and the data management plan. Furthermore, researchers often need to work within challenging contexts, as is the case in tuberculosis services, where human and technological resources for research may be scarce. Electronic Data Capture Systems mitigate such risks and enable a reliable environment to conduct health research and promote result dissemination and data reusability. The proposed solution is based on needs pinpointed by researchers, considering the need for an accommodating solution to conduct research in low-resource environments. The REDbox framework was developed to facilitate data collection, management, sharing, and availability in tuberculosis research and improve the user experience through user-friendly, web-based tools. REDbox combines elements of the REDCap and KoBoToolbox electronic data capture systems and semantics to deliver new valuable tools that meet the needs of tuberculosis researchers in Brazil. The framework was implemented in five cross-institutional, nationwide projects to evaluate the users' perceptions of the system's usefulness and the information and user experience. Seventeen responses (representing 40% of active users) to an anonymous survey distributed to active users indicated that REDbox was perceived to be helpful for the particular audience of researchers and health professionals. The relevance of this article lies in the innovative approach to supporting tuberculosis research by combining existing technologies and tailoring supporting features.

## Introduction

Data collection is one of the most crucial aspects of any research project, as it can determine its success or failure. The lack of quality data is sometimes noted during or after the collection phase. In order to avoid this, it is essential to implement a reliable data capture system, in addition to employing a trained data collector^1^.

Additionally, the success of clinical research directly depends on the correct definition of the research protocol, the data collection strategy, and the data management plan^2^. These elements drive the quality and reliability of the collected data that will be used to analyze the outcomes of a given study.

Adopting new methods, tools, and data sources has changed how research is conducted. However, new challenges have arisen, demanding innovative approaches to collecting, managing, and publishing data. Well-managed data are easier to use and analyze to confirm a research hypothesis. Also, the reuse of data in further studies is enhanced. In other words, it stimulates research collaboration and maximizes funders' investment^3^.

Electronic Data Capture (EDC) systems are valuable tools for clinical trials and research to capture data^4^ and should offer improved data integrity, cost savings, and a shorter time to study database closure^5^. They may help researchers comply with medical regulations and enhance data quality and researchers' management capability^6^. Preferably, EDC systems should also be interoperable so they can communicate with other systems and promote seamless data exchange and integration^7^.

Research Electronic Data Capture (REDCap)^8^ and KoBoToolbox^9^ are two well-known EDC systems. The first one presents a better approach to the whole research life cycle but has significant disadvantages, such as usability issues and the inability to working offline without additional software. The second one delivers a user-friendly interface and natively works offline through a mobile browser but has limited features for data management.

Moreover, in health research, researchers must work in different environments, ranging from facilities with high-tech devices to those with limited access to resources, such as poor or no internet connection or lack of reliable electrical power.

In the case of tuberculosis (TB), an infectious and neglected disease^10^, the resources for research may be lacking, and the costs of using an EDC could be a limitation. The scenario is aggravated by the fact that Brazil is among the top 30 countries with the highest TB burden^11^. These aspects stand out as barriers to collecting data in TB research. Therefore, making data available for further data-driven studies is crucial to underpinning the development of new evidence-based decision-making tools.

Integrating information into more extensive systems is hampered by data formats and structural heterogeneity. Data must be correctly described in order for it to be beneficial^12^. Thus, semantic interoperability is a critical consideration in information system design^13^. It is achieved when one system can understand the context and meaning of the information provided by another system^14^.

Meaning can be imparted to data by using ontologies or other semantic standards, i.e., well-defined vocabularies that allow a precise and machine-readable description of domain-specific knowledge^15^. It may enable semantic interoperability, allowing systems to interpret the data in accordance with its formal definition^16^. In this sense, data can be shared accurately and reliably to enhance communication among computerized systems. This capability is especially desirable in health information systems (HIS) due to the heterogeneity of the medical language and health-related concepts^14^.

Ontologies are essential in semantic alignment for data integration, information exchange, and semantic interoperability^17^. An ontology comprises several properties, each describing a specific piece of data in the domain being represented^18^. Besides ontologies, simple standards such as the Humanitarian Exchange Language (HXL) help speed up data processing and create interoperability across data sources. HXL is a project by the United Nations Office for the Coordination of Humanitarian Affairs to coordinate disaster response using semantic web technologies. It uses simple marking through hashtags and aims to contribute to automating processes to improve information flow to decision-makers^19^.

In the case of health research, semantic annotation can help describe the data that is being collected. It can be helpful to extract and link different research datasets described using the same vocabulary. Usually, each study consists of several collection instruments, totaling hundreds of fields to fill during the research process. Manual annotation is a valid choice for semantic annotation, but automated approaches are preferable^20^.

## Objectives

This manuscript presents REDbox, a comprehensive framework based on the REDCap^8^ and KoBoToolbox^9^ systems. The authors of this manuscript developed REDbox to enhance research data collection and management in TB services, as well as in similar low-resource research environments in Brazil while providing a better user experience.

Additionally, REDbox promotes the semantic interoperability of research data. Relying on ontologies and HXL to perform semantic annotations, the objective is to automate the design of an instrument based on a given ontology and the generation of ontologies derived from the instrument's schema, as well as to increase the availability of data for further data-driven TB research.

## Methods

In this research, the authors used no clinical data nor private or public databases to conceive and develop REDbox. All methods were carried out following relevant guidelines and regulations. Therefore, no ethical approval was necessary. This section details the scientific method and the essential technological tools upon which this work is based.

### Solution development and validation

The basis for this work is action research. It is a suitable methodology because it simultaneously assists practical problem-solving, expands scientific knowledge, and enhances the respective actors' competencies^21^. Considering that the research has a practical component in addition to its theoretical development, action research appears to be a good approach.

Action research is an interactive inquiry process that balances problem-solving actions implemented in a collaborative context with data-driven collaborative analysis or research to understand underlying causes, enabling predictions about future personal and organizational change^22,23^. The research started with identifying the research goal: a framework to support research in the challenging conditions of TB services in Brazil. The first step, ideation, provided a starting point. After conducting a literature review and identifying existing frameworks and tools, it was possible to identify challenges and unresolved issues. It was then possible to evaluate the research questions and refine them based on prior research. A thorough reflection on the problems and possible solutions through an iterative process involving researchers in the field pinpointed vital issues and ways to tackle them. Concretization of solutions involved cycles of analysis, reflection, and feedback.

In this sense, REDbox modules were developed by analyzing the primary needs reported by researchers and research teams with considerable experience in TB. The authors participated in several interactions with independent teams to build the framework based on the REDCap and KoBoToolbox tools, which were identified as valuable assets in scientific research. With that in mind, REDbox was developed to fill in the gaps left by these tools and allow researchers to work seamlessly with these platforms.

**Figure 1:**
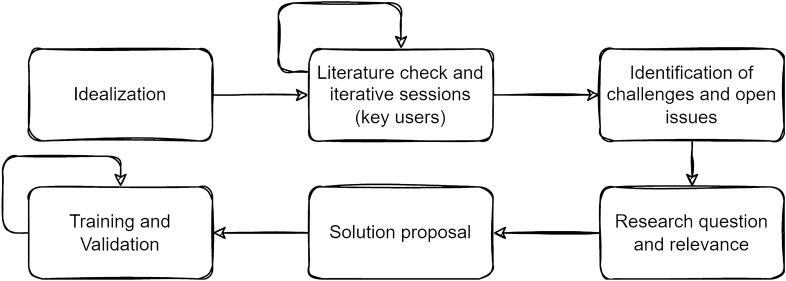
The scientific method.

Therefore, this research comprises steps to adequately identify the challenges and open issues regarding the computational tools available for data collection, management, and sharing in low-resource environments. Considering the theme's relevance, the research questions were defined to guide the solution proposal. Finally, the validation phase was performed through a field test covering user training and satisfaction analysis. Figure 1 summarizes the scientific method.

The primary needs were analyzed in the field through an iterative and interactive process, as shown in Table 1. The coordination staff (e.g., health managers, health professionals, and physicians) of TB services were defined as key users and actively pointed out their recurrent needs regarding human and technical resources, data availability, and patient safety. These users are relevant focal points because they can provide their opinion based on their long-term experience and comprehensive knowledge of TB research and care services in Brazil.

**Table 1:** Identified needs.

Type of need	Description
Technical resources	• Poor internet connection • Unavailability or outdated devices
Human resources	• Staff with low familiarity with digital tools
Research data	• Low availability (lack of sharing) • Low quality
Patient safety	• Ensure an efficient, effective, and safe service for patients • Good clinical practice

Some obstacles make it difficult to make data available, such as using non-standardized vocabularies/terminologies, using legacy systems, and the enormous bureaucracy involved in accessing health data. Although complex, sharing health data can enhance research activities and increase a health service's clinical and operational effectiveness^24^. Data sharing requires functional and semantic interoperability capabilities to properly communicate and understand the data^25,26^.

Therefore, the need for an accommodating option to conduct research and promote data sharing in TB services led to the conceptualization of the open-source solution proposed in this work. There was none found in the literature, and after rounds of discussions with researchers, developing a module-based and customized software to overcome existing technological barriers in TB services was defined as the main challenge to overcome.

Therefore, the following research questions were defined:

- “Would the development of a tool for collecting and managing research data be useful for researchers in TB services and similar low-resource environments?”
- “What can be done to promote data interoperability and improve the availability of tuberculosis data for researchers?”

The solution is relevant because it may:

1. Improve the collection and analysis of research data during the whole study period;
2. Facilitate the management of research events and data;
3. Increase the user experience by combining positive aspects of existing solutions;
4. Increase the security of research data;
5. Remove technological barriers by delivering an approach that works on any device and without an internet connection;
6. Remove cultural barriers, such as the lack of confidence of researchers to drop paper-based methods;
7. Promote semantic interoperability for data reuse and record linkage.

The solution has research teams, research centers, and study participants as stakeholders. Targeting their needs and identified challenges (see Tables 1, 5), REDbox delivers useful functionalities for the collection and management of research data and promotes the availability and reusability of research data.

For the validation phase, the modules developed by the authors were used in five cross-institutional TB research projects in Brazil (see Table 3). Also, it is demonstrated how semantics can promote the reusability and interoperability of research data.

### REDCap and KoBoToolbox as electronic data capture systems

REDCap is a web-based, metadata-driven software built in 2004 by a team at Vanderbilt University to enable classical and translational clinical research, basic science research, and general surveys, providing researchers with a tool for the design and development of electronic data capture tools^8,27^. REDCap is free, but it is not considered open source. A license is required to operate it, and it can be installed and managed by a small IT team^28^. In the context of this work, a REDCap is maintained by the Brazilian Network for Tuberculosis Research (REDE-TB) was used (available at https://redcap.redetb.org.br/, version 13.4.13).

KoBoToolbox is a free, open-source suite of tools for data collection and elemental analysis developed by the Harvard Humanitarian Initiative. It was initially built for use in challenging environments in developing countries^9^. KoBoToolbox is powered by the Enketo open-source project^29^ and offers online and offline functionality and is accessible from any modern browser thanks to HTML5 features. The software relies on the XLSForm standard, which simplifies the authoring of forms in spreadsheets in a human-readable format^30^. A visual and intuitive form builder is available, or forms can be imported as XLS files.

The scientific community widely uses the REDCap system to collect and manage research data, allowing researchers to conduct their studies independently. However, the software may present some usability issues during data collection, such as a polluted graphical interface, gradual performance degradation, and the lack of offline operation without depending on a mobile application.

Although it presents basic functionalities, the KoBoToolbox delivers modern styles and allows users to work offline directly from the web browser. Therefore, the software may be an essential component in mitigating the usability issues of REDCap.

### Data annotation for semantic interoperability

To better represent collected data, fields in research forms can be annotated with semantic vocabularies. REDCap offers the possibility to include annotations for each field, which will not be displayed on the form or survey but will be available to the designer and in data exports to help understand the data^27^. This annotation can be a property of an ontology or an HXL hashtag, depending on the user's preference.

KoBoToolbox natively supports the use of HXL. When authoring an XLSForm, the user must insert one extra column in the spreadsheet and fill it with HXL hashtags identifying the type of information in each column. The form builder also provides an intuitive way to relate a hashtag to an instrument's field.

## Results

**Figure 2:**
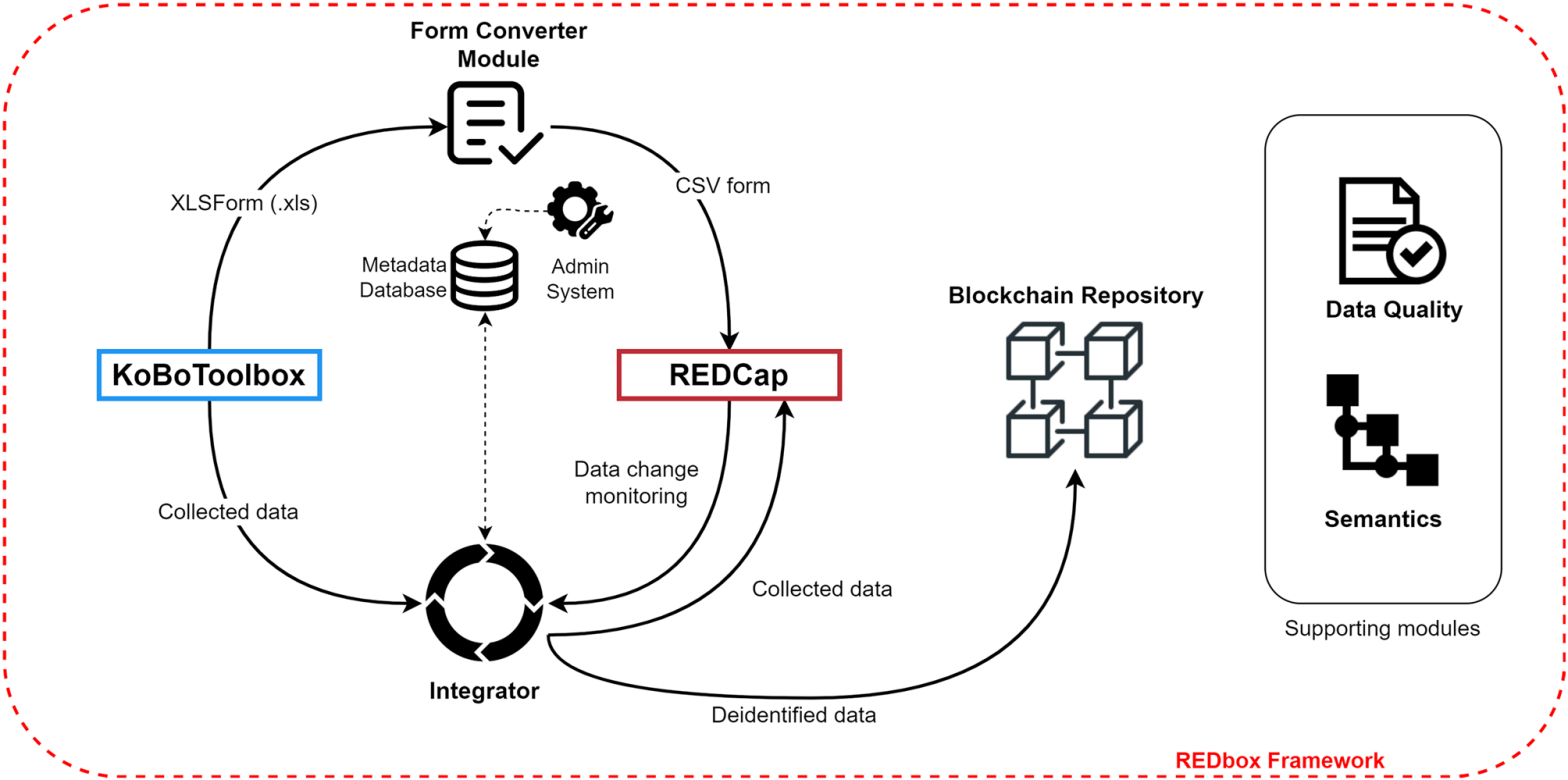
REDbox framework overview.

The framework was developed using the PHP v7.4 scripting language^31^ and is composed of five modules, which are as follows: (i) a metadata database and an Admin System; (ii) a Form Converter; (iii) an extract-transform-load (ETL) processor; (iv) a Data Quality Module; (v) and the Ontology Services. Figure 2 shows the REDbox framework overview.

### The metadata database and the Admin System

**Figure 3:**
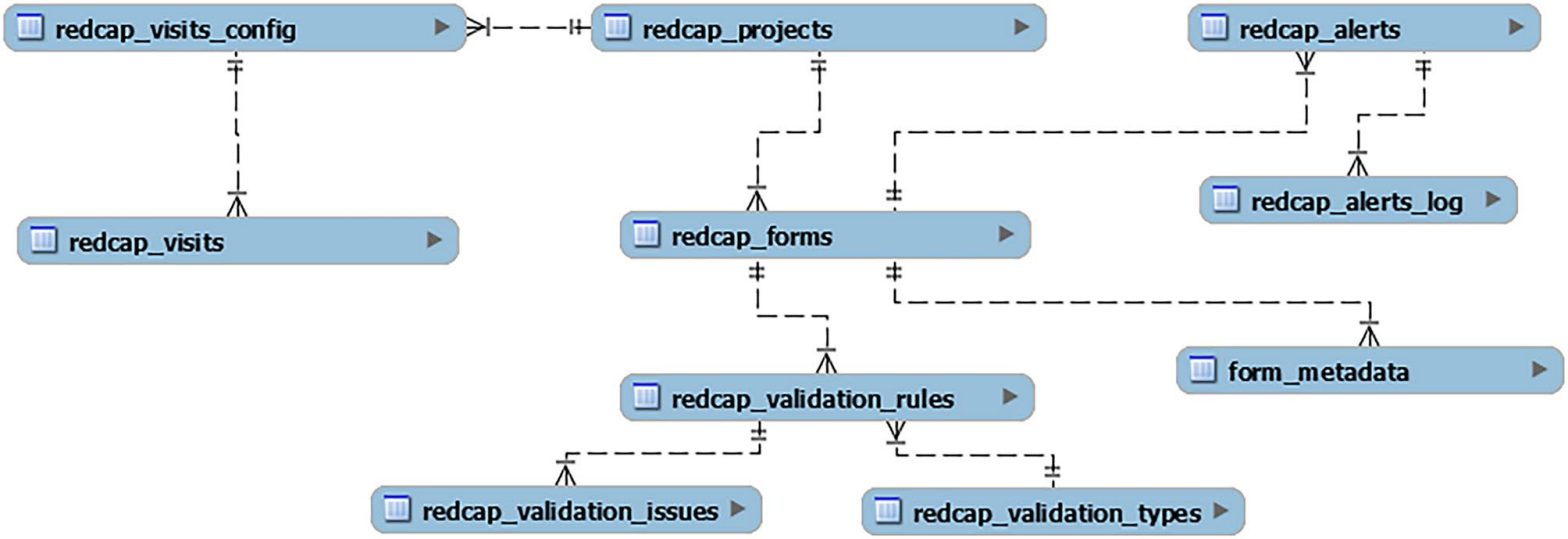
Relational database model.

The web-based Admin System was developed in C#^32^ and JavaScript^33^ programming languages to efficiently manage the mandatory metadata through create-read-update-delete (CRUD) operations. Figure 3 presents the relational model (database tables and relationships).

In general, an entry must be created in *redcap_project*, the main table that stores descriptive information and REDCap's Application Programming Interface (API) credentials. Then, each project's instrument must be registered in *redcap_forms*. The *form_metadata* stores semantic mapping for the instrument's fields. Additionally, the Data Quality Module relies on the following tables in the database: *redcap_validation_types*, *redcap_validation_rules*, *redcap_validation_issues*, *redcap_visits*, *redcap_visits_config*, *redcap_alerts*, and *redcap_alerts_log*.

### The form converter

Since instruments are built using specific standards in each software, a converter is desirable, so the designer does not have to create the exact form twice. This module allows forms in REDCap to be automatically created through ontological derivation or by converting a form designed to the XLSForm standard, as described below.

**Figure 4:**
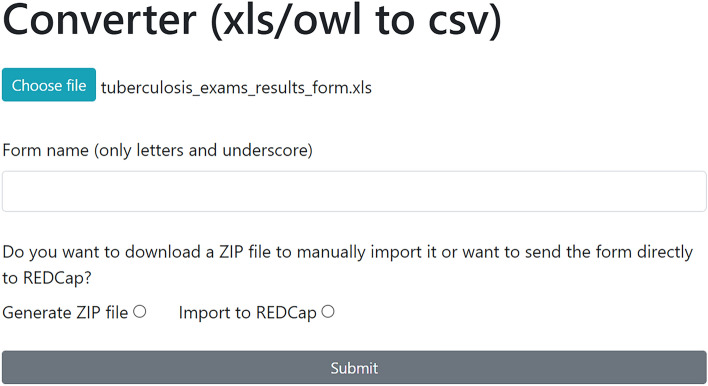
Converter module—user interface.

To initiate the process, the user must upload the spreadsheet (.xls) or the ontology (.owl) file, fill in the form name, and choose between generating a .zip file, manually uploading it into REDCap, or automatically importing the form through the API. In the second option, the API token and URL must be provided. Figure 4 shows the user interface of the converter.

*Deriving from ontologies.* Each property of a given ontology can be converted to fields in forms. The name and type of a field are obtained from the name of the property and the associated type (text is the default type). Minimum and maximum values defined as restrictions on properties are also converted.

*Converting from XLSForms.* The converter supports all common field types, such as text, date, date and time, time, integer, decimal, calculation, single selection, multiple selection, files, and notes. These fields, including the variable name and values assigned to options in single and multiple selections will be converted as-is so that instruments on both systems will have a matching structure. Skip logic defined on KoBoToolbox is translated to REDCap branching logic and validation rules.

In the designing process, there is a particularity related to multiple-selection questions (checkboxes). This type of question must have the field's name starting with "*checkbox_*". This convention is needed to ensure the correct identification of a multiple-selection question structure during data transfer from KoBoToolbox to REDCap.

Before starting the conversion process, the converter module will pre-check the naming convention. If any inconsistency is detected, the conversion will fail, and the user will be informed of the error.

### The ETL processor

After converting the instrument and transmitting it to REDCap, KoBoToolbox native REST Services must be enabled in the form settings to instantly submit collected data to the ETL processor through a POST request. The processor URL and basic HTTP authentication credentials must be provided.

The Processor receives the data collected in KoBoToolbox as a JSON object, parsed to remove unnecessary elements unrelated to the data of interest. After verifying the authentication credentials, the metadata is queried to obtain the URL and the token of the REDCap API (from *redcap_projects*) and to verify if it is the first form in the project (from *redcap_forms*). If it is, a request is sent to the REDCap API to generate a new record ID, which means it is a new participant in a research project. Otherwise, the record ID will be searched in the log of collected data based on the participant identifier. Then, a request is sent to the REDCap API to import the data.

After successfully saving the data, additional steps may take place depending on the settings defined for the instrument. Sending email notifications (both for the respondent and the research team), verifying the duplicity of records, and the instant lock of the saved record (to avoid changes in the data) are possible extra actions. These are valuable features that facilitate the management of research data.

Once the data is in the REDCap database, changes in records are monitored through the Data Entry Trigger module, which can detect any changes. When it occurs, the Processor exports the edited data from REDCap and logs it into the relational database.

### Data quality module

Data management is a continuous process and represents a critical phase in clinical research due to its importance in generating high-quality and reliable data for statistical analysis, which must meet the protocol-specified parameters and adhere to research protocol requirements^34^.

The management activities must occur in parallel with the data collection. The data manager usually carries out a data validation process, which includes the verification of the consistency, completeness, and accuracy of collected data. This is expected to prevent data loss and increase quality.

In health research, most data are acquired during participant visits. Therefore, keeping track of the schedule of visits and their status (carried out, not carried out, pending) is essential for achieving all milestones.

However, all of these tasks are time-consuming because they demand the careful inspection of a significant amount of data. The REDCap software natively offers valuable tools to help data managers and researchers. As examples, the Resolution Workflow and Scheduling features allow the opening of queries to request the verification of the collected data and assist in scheduling expected visits for participants during the study (although it requires a manual setup for each participant), respectively.

The Data Quality Module is composed of six submodules that complement the functionalities offered by REDCap, focusing on the reduction of the workload for data managers and researchers, namely: Data Validation Rules, Events/Visits Calendar, Alert System, Instruments Validation, Data Management Plan Creator, and User Support.

**Figure 5:**
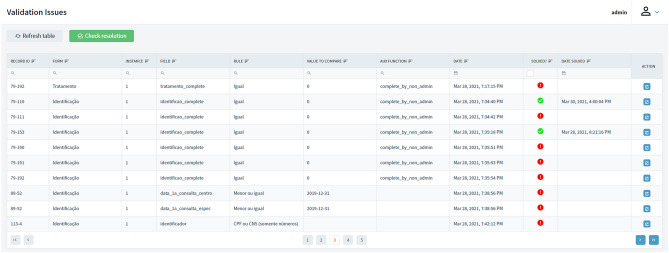
Data Quality Module—validation issues dashboard.

First, an automatic rule-based validation procedure searches for inconsistencies through each field in all instruments. Rules must be pre-defined in the form of metadata and represent the format or range of values expected for a given field. The procedure runs several times a day, at the same time, to check for new issues and verify the resolution of previously identified ones. When an issue is detected, a query is opened in the Resolution Workflow (in REDCap), and the data collector is alerted by email. Figure 5 presents the dashboard with an overview of all issues detected in a REDCap project.

**Figure 6:**
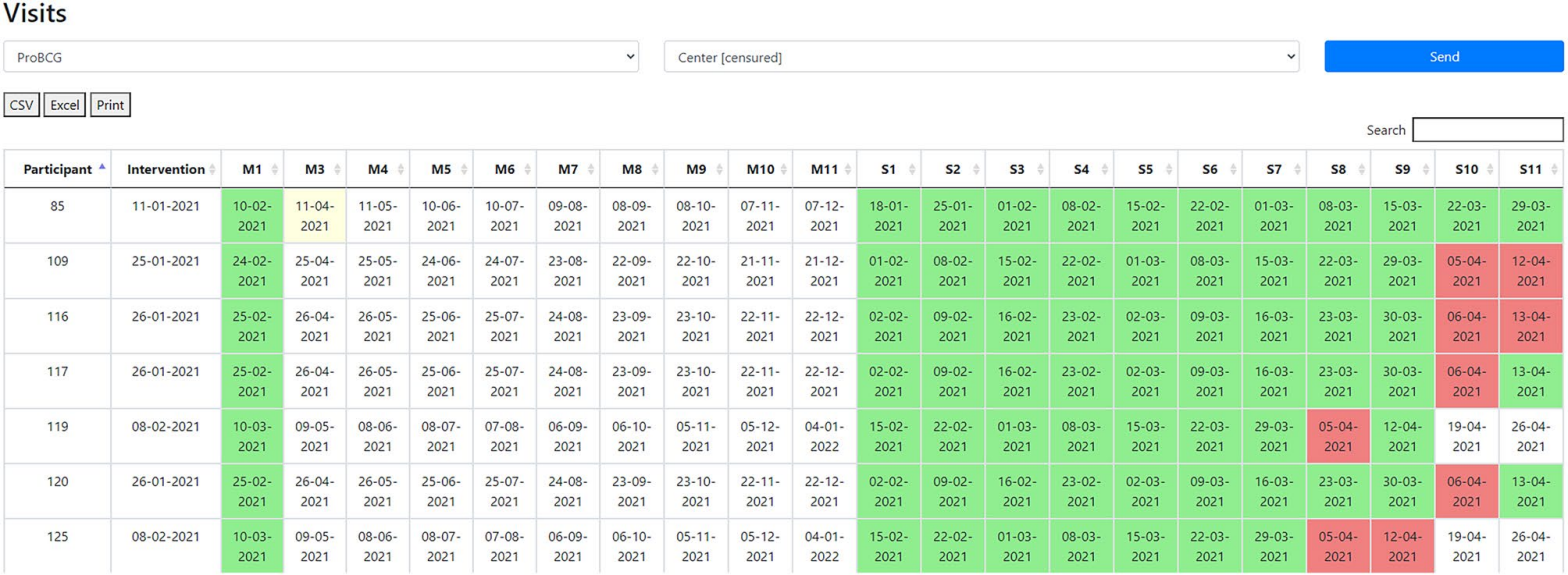
Data Quality Module—visits panel.

The Events/Visits Calendar is a panel that visualizes all upcoming participants' visits. Each row in the panel is a participant, and each column is a visit. The color of the cells represents the status of a visit (green: carried out; red: not carried out; yellow: pending/waiting for the participant). Dates are calculated based on a reference date field (e.g., the day of an intervention or inclusion in the study) and the days offset for each event. This information is also stored as metadata. The panel is created in real-time with online data extracted from the REDCap database, saving time for researchers who usually create their panels using spreadsheets. Figure 6 shows the panel for a study with 21 visits (project IV in Table 3).

The Alert System was designed to periodically send notifications to the research centers regarding not-answered queries and pending data collection based on the scheduled events of each study. Through these reminders, the system helps researchers keep participants' data up-to-date according to the formal protocol, avoiding critical protocol violations. The notifications may be sent by email or SMS to the recipients' lists stored as metadata.

**Figure 7:**
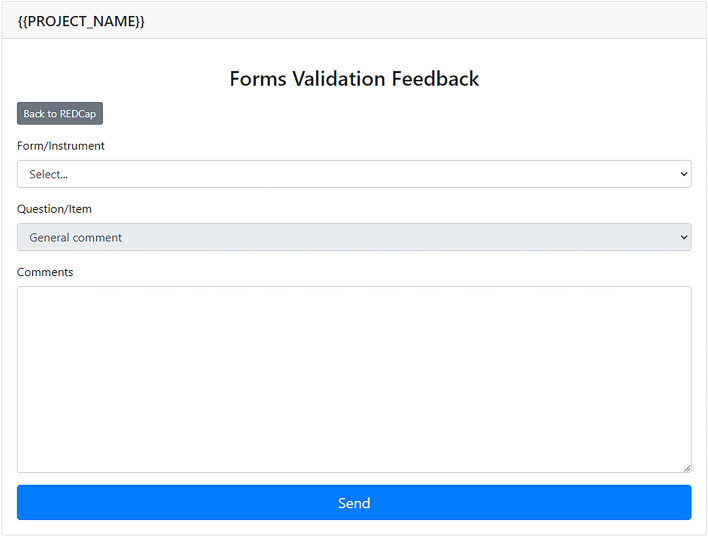
Instruments Validation—commenting interface.

The Instrument Validation module allows the research team to comment on the data collection forms and exchange insights in a centralized platform. In this sense, the discussion focuses on specific aspects of each instrument pointed out by team members, allowing researchers to identify and address possible design errors quickly. After obtaining comments from invited users, the coordination staff can start discussing them (via replies to the original comment) through an administrative web interface. Figure 7 presents the commenting interface where users can provide feedback about the instruments and the questions.

The Data Management Plan Creator (https://redbox.technology/pgd.php) is a web tool that allows users to quickly create a complete plan based on a document model established in collaboration with several researchers. This model contains all the essential content a data management plan must have, such as information about the study data and resources, security and privacy, ways of accessing and archiving data, and ethical and regulatory aspects. The user must fill out some metadata fields (project name, project number, principal investigator name, funding, among others), and a Google document (the only requirement is to own a Google Account) is instantly generated with the user as the owner. The resulting document can be manually edited and adapted to a specific project. The data management plan is generated only in the Portuguese language.

**Figure 8:**
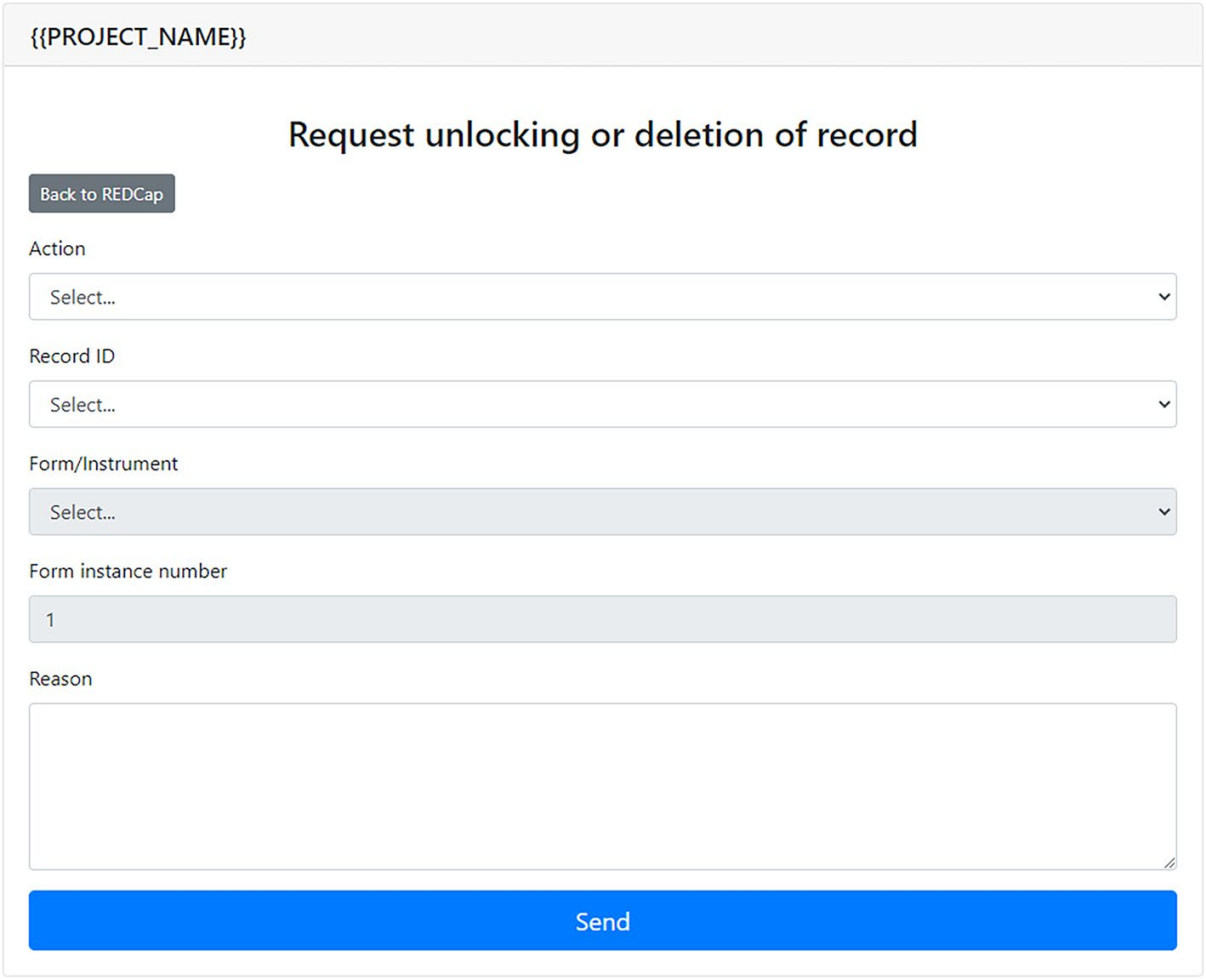
User support interface.

Finally, the User Support module is a supporting tool to facilitate communication between research teams (often located in distinct research centers) and the project's coordination staff (Fig. 8). This tool allows users to send specific requests regarding the data stored in the REDCap database, such as unlocking records for editing and data deletions. A detailed log of all requests is maintained for accountability purposes.

### Ontology service

The solution offers a service that provides practical tools to enhance the use of ontologies in the system and allow the continuous integration of different data sources, adapt to the evolution of ontologies, ensure availability, and avoid data loss.

As previously stated, the form converter can derive an instrument from an ontology. Similarly, this service enables the creation of an ontology based on an instrument. This feature relies on an external application, the D2R Server^35,36^. The D2R is a tool that converts relational content into semantic formats, allowing a quick conversion between these formats by automatically creating ontologies based on the schema of the content.

Relying on this feature, REDbox can define an ontology from a data collection instrument. For this, a temporary table is created on a relational database, where each column represents a field in the instrument. Then, the D2R generates and publishes an ontology using the table structure, i.e., converting columns to properties, which can be later customized. Table 2 presents an example of an ontology generated from an instrument containing a patient's treatment data.

**Table 2:** Instrument and ontology correspondence.

Instrument		Ontology	
Field	Type	Property	Range
Start date	A textbox with date validation	http://vocab.redbox.technology/vocab/treatment/start_date	Literal (date)
TB clinical form	Multiple choice with a single answer	http://vocab.redbox.technology/vocab/treatment/clinical_form	Literal
Discharge date	A textbox with date validation	http://vocab.redbox.technology/vocab/treatment/discharg_date	Literal (date)

The Ontology Service guarantees semantic interoperability between the applications and formularies that use different versions of the same ontology or even between different ontologies by maintaining the history of changes and mapping the concepts from one ontology version to another. This service accepts annotated files with an ontology version that can be converted to an older or newer version of the same ontology and annotated files to be converted to a correlated ontology (in the latter case, a prior mapping of ontology properties as metadata is required).

### System flow

**Figure 9:**
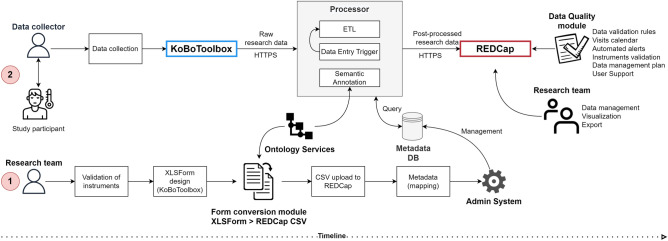
System and data flow.

All REDbox framework modules work in an integrated way. In a research project's initial phase, two paths must be followed to execute the planned activities seamlessly. Figure 9 represents the system and data flow.

The first path (red round label with “1”) refers to the pre-collection phase. The research team must proceed with developing and validating the collection instruments. These are crucial activities for defining the types of data and formats needed and the collection strategy. It must be carried out carefully with the participation, preferably, of representatives of all research centers involved in the project.

Then, the instruments must be designed in the KoBoToolbox (generally, using the native builder)—the result is a form in the XLSForm standard—and converted to the format accepted by REDCap Comma Separated Value (CSV) through the Form Converter module. The resulting file must be uploaded into REDCap. Finally, the metadata of each form must be mapped, namely the fields that contain personal identifiers and the semantics (e.g., ontologies) potentially associated with each field.

The second path (red round label with “2”) refers to the collection and continuous research data management process. Data are collected during the interviewer's interaction with the research participant through the form available in the KoBoToolbox system. After that, the Processor extracts, transforms, and processes the data for later storage in the REDCap database. The Processor also monitors possible changes in the data through the Data Entry Trigger, offering flexibility for new processing while enhancing the security of the research data.

Finally, using additional tools provided by the Data Quality module (validation rules, calendar, alerts), the research project team can manage the data and follow the project during the research lifecycle. Data can be visualized and exported directly from the REDCap system.

### Validation

The validation of the proposed solution is performed by using it in several cross-institutional research projects related to TB in Brazil, namely: (I) Longitudinal Study of the Impact of Social Support on Tuberculosis Indicators: ELISIOS; (II) Validation of the Line Probe Assay's performance as a rapid diagnostic method for drug-resistant tuberculosis in reference centers in Brazil; (III) Validation of Recombinant PPD in the Diagnosis of Tuberculosis Infection; (IV) ProBCG: Use of the Bacillus Calmette–Guérin (BCG) vaccine as prevention of COVID-19 in health professionals; and (V) Multicenter prospective clinical trial to assess the diagnostic accuracy of the Truenat method for routine use. Table 3 shows the characteristics of each project currently using the framework.

There are a significant number of instruments and fields on each project. The form converter module is crucial in this scenario, where each form must be designed only once in KoBoToolbox and then converted to the REDCap format. The expected number of records is also significant, which may demand easy-to-use and offline tools.

So far, the main benefits reported by end users of research centers relate to the ability to collect data in interviews with patients in scenarios with an unstable internet connection, receive personalized alerts based on events, and the possibility to quickly visualize the expected visits of each participant during the study.

Besides frequently evaluating the feedback received through interactions with key users to guide the development and improvement of the REDbox framework, registered and active users (from the projects presented in Table 3) were asked to complete a usability and satisfaction questionnaire to verify their perceptions regarding information quality, interface quality, and system usefulness.

The IBM Computer System Usability Questionnaire (CSUQ) version 3^37,38^ was applied to assess the general satisfaction of users regarding the modules and functionalities available^37,38^. It is an easy-to-use instrument with 16 questions that use a 7-point psychometric Likert scale, strongly agree (1) to strongly disagree (7), to measure human attitude^39^, and assess perceived usability. The CSUQ produces four scores (in this case, higher is better), one overall and three subscales^40^, as follows:

- Overall: average responses for questions 1–16 (all)
- System Usefulness (SysUse): average responses for questions 1–6
- Information Quality (InfoQual): average responses for questions 7–12
- Interface Quality (IntQual): average responses for questions 13–15.

Seventeen responses were collected, representing 40% of the active users. Of this total, seven (41.18%) respondents were men, nine (52.94%) were women, and one (5.88%) did not provide an answer to the question about their gender. The respondents' ages ranged from 21 to 49 years. The job position was also asked and distributed as follows: nurse (2), physician (5), system analyst (1), student (2), researcher (2), project fellow (3), laboratory technician (1), and not answered (1).

CSUQ can be used with larger sample sizes (more than 100) and smaller ones (fewer than 15). Despite the difference in precision, according to Tullis and Stetson, a sample size of 12 generates the same results as a larger sample size 90% of the time^41^. Yet, small samples are typically seen in usability and satisfaction tests and are generally sufficient for usability evaluations^42,43^. Therefore, the number of responses obtained is satisfactory.

**Table 3:** Characteristics of each project that is currently using REDbox.

Project	No. of research centers	No. of instruments	No. of fields	Expected no. of records
I	3	4	175	2500
II	12	14	679	3800
III	10	9	183	1020
IV	3	24	528	1000
V	5	14	357	500
Total	32	65	1919	8820

The participation of users was wholly voluntary and anonymous. Knowing the respondents' identities would not be useful in this phase, so only the minimum data was collected. A public survey link was made available and is the same for everyone, not allowing authors to track respondents. However, due to the anonymity of the survey, it could not be restricted to single participation (one response per user). To mitigate this, we provided instructions in the invitation email that multiple responses should not be sent.

It was a participatory activity to engage users in the system and providing feedback regarding information quality, interface quality, and system usefulness. Table 4 presents the response averages to the 16 questions and the four calculated scores. It is important to note that the questions do not refer to specific modules but aim to assess the general perception of the REDbox framework.

**Table 4:** Response averages to CSUQ Questionnaire questions.

#	Question	Average	Subscales	Overall
1	Overall, I am satisfied with how easy it is to use this system	6.25	SysUse 6.26	6.02
2	It is simple to use this system	6.19		
3	I am able to complete my work quickly using this system	6.44		
4	I feel comfortable using this system	6.38		
5	It was easy to learn to use this system	6.06		
6	I believe I became productive quickly using this system	6.25		
7	The system gives error messages that clearly tell me how to fix problems	5.19	InfoQual 5.76	
8	Whenever I make a mistake using the system, I recover easily and quickly	5.38		
9	The information (such as online help, on-screen messages, and other documentation) provided with this system is clear	5.63		
10	It is easy to find the information I need	6.00		
11	The information is effective in helping me complete my work	6.44		
12	The organization of information on the system screens is clear	5.94		
13	The interface of this system is pleasant	5.94	IntQual 5.96	
14	I like using the interface of this system	6.00		
15	This system has all the functions and capabilities I expect it to have	5.94		
16	Overall, I am satisfied with this system	6.25		

## Discussion

The relevance of this article lies in the innovative approach to supporting TB research. The REDbox framework offers valuable tools and a better user experience by integrating the REDCap and KoBoToolbox EDC systems and using semantics. The proposed solution facilitates the collection and management of research data. Despite being based on the TB context, the framework can be applied in other contexts with the same demands.

The primary motivation for this work was to allow health research to be carried out in TB services, where, in general, technological resources are scarce and precarious. Considering that the cost of an EDC system is high, the monthly or annual payment of licenses harms the research budget. Yet, as alternative solutions, REDCap and KoBoToolbox do not meet all the researchers' needs. Therefore, the gaps filled by the REDbox framework represent a significant advance in the free tools available for research.

The CSUQ questionnaire allowed the authors to verify the overall satisfaction of active users and the System Usefulness, Information Quality, and Interface Quality subscales. Even though it got a small number of responses, it still represents a good portion of the active user database. As already mentioned, despite the difference in precision between larger and smaller samples, the results obtained through the application of the CSUQ are valid for small samples of usability and satisfaction tests.

Overall, the average demonstrated that users are mostly satisfied with the system. Also, the three subscales performed above the middle range (3.5) of the 7-point Likert scale and showed that the system has room for improvement. The InfoQual and IntQual subscales may show that the information should be better organized. The system could be improved in terms of communication and interaction with the user, and the user interface could be more friendly and intuitive. However, SysUse demonstrated that the system fulfills the function for which it was designed, and that is, in fact, efficient and effective for end users.

Although the CSUQ questionnaire does not point out precisely what REDbox's shortcomings are, it is noted that users still have the perception that some points can be improved, even with the good performance indicated by the information and interface quality metrics.

However, it is possible to speculate on aspects that can be improved in future software versions, including providing more accurate information, handling errors and on-screen messages, improving the usability and responsiveness of graphical interfaces on mobile devices, the provision of user manuals, and creating new features. Once the system's deficiencies are overcome, a higher score is expected in future usability and satisfaction tests.

### Implementation and requirements aspects

Although a REDCap mobile application^44^ is available to enable offline data collection, more may be needed due to the dependency on smartphones and/or tablets available in research centers, the poor usability, and the non-compatibility of some advanced features^45^. Also, mobile devices in digital data collection projects are frequently not owned by the people entering the data, which can be considered a risk to be managed^46^. On the other hand, due to the use of HTML5 features, KoBoToolbox provides a better user experience through modern form styles and a way to work offline, if needed, without using any additional applications, such as mobile apps.

Benefits are added for both EDC systems, and the user/researcher can take advantage of the best of each system. In this sense, the negative aspects of one can be mitigated by the positive characteristics of the other. REDCap is effective and efficient for managing data and conducting research after the initial collection phase, allowing researchers to have a more comprehensive view of the project database, including creating custom reports and accessing descriptive data analysis. KoBoToolbox allows for a more delightful collection through a clean, friendly, practical, and accessible interface for any device. Then, the REDbox framework fills the remaining gaps by offering extra functionality to enhance the researcher experience and underpin the research data lifecycle. The Table 5 summarizes the requirements of each stakeholder.

**Table 5:** Requirements for each stakeholder.

Stakeholder	Challenges	Solutions
Individual researchers/teams and research centers	• Availability of quality data for TB data-driven studies • Reduced technological and human resources • Lack of a royalty-free and comprehensive solution	• Promotion of data interoperability and sharing • Promotion of the use of semantics • A free and open-source solution • Availability of several tools and functionalities for better data collection and management • Reduced workload
Study participants	• Safe interventions • Guarantees of respectful and adequate use of personal data	• Events/visits scheduling • Careful data handling and management • Adverse events monitoring • Mitigation of human errors

*Semantics.* Semantic annotation can underpin the exchange, use, and integration of data from different sources thanks to the aggregation of meaning in raw data. In other words, data becomes machine understandable and can be interpreted by distinct systems.

In research project IV, as shown in Table 3, a semantic integration has been performed using data collected by the research's instruments and HIS from the Brazilian Ministry of Health. In this case, demographic and vaccination information were integrated and compared to keep the data up-to-date and increase the completeness of the research dataset.

Although the solution does not perform or implement semantic interoperability mechanisms, it focuses on adding meaning to data to support semantic data integration and interoperability based on standards, vocabularies, and ontologies. Standard EDC systems usually do not present this type of feature.

Users can prepare their research datasets to be shareable, reusable, and understandable. The ability to map variables when creating data collection instruments is a breakthrough because the dataset will be fully annotated (with semantics) when the collection phase is over. Therefore, regardless of how the data is exported and shared, the researcher will always have the option of including the semantics associated with the data.

The other possibility is also valid and helpful, as it can reduce the workload of the research teams associated with creating data collection instruments. The derivation of a set of variables and forms from an ontology facilitates the visualization of what should or must be collected in a given knowledge domain, reducing the occurrence of human errors and also automatically aggregating meaning to data.

*Application Programming Interfaces—APIs*. APIs enable interoperability and data integration between software components and the development of extensions to existing systems.

Regarding REDCap, the API is well documented, and several endpoints are available, allowing for programmatically managing a whole project. In this work, some endpoints were used, specifically to: i) import and export data; ii) import files; iii) generate unique identifiers (record IDs); iv) import metadata (instruments, fields); and v) export metadata.

In KoBoToolbox, the API must be adequately documented. However, there is a feature to instantly send collected data to an external server (data is sent in JSON standard). This feature is handy when using the system only for data collection, which is the intention of this work, and because it eliminates the need to develop a client to extract data.

*Data safety*. Data is generally stored in three distinct logical units: the KoBoToolbox, REDCap, and relational databases. Only the data stored in REDCap is intended for analysis, but data can be quickly restored in the event of a failure. Finally, the whole process is transparent to the final user, who can focus only on data collection, management, and analysis.

*Limitations*. In the form converter, the designer must pay attention to the following aspects:

(i) *need to use a variable naming convention for multiple selection fields (checkboxes).* Using a naming convention for variables in multiple selection fields is crucial. Otherwise, data transfers may fail.
(ii) *calculated fields.* When using calculated fields, KoBoToolbox does not allow setting up a label for this kind of field, unlike REDCap. The designer can use the "Guidance Hint" option as a workaround, which will be transformed into a label when converted to REDCap format. However, this is optional since REDCap accepts blank labels in calculated fields.

A drawback of using the REDbox framework is the need to define several configuration parameters in the metadata database for the proper functioning of the system and the effective integration of REDCap and KoBoToolbox. It may represent a workload in the initial phase of the research project (the setup must be carried out before starting the data collection), which varies according to the modules used. The Admin System and user's manual seek to make this task more accessible, but some technical knowledge may be necessary for a correct configuration.

## Conclusions

This work has presented REDbox, a comprehensive framework for integrated data collection and management in tuberculosis research. The use of REDCap and KoBoToolbox together has allowed the transparent combination of the advantages of each, helping researchers manage and maintain data while increasing the satisfaction of the final users responsible for collecting data in the field.

The Form Converter avoids rework in defining variables/fields and designing data collection instruments. The ETL Processor enables data transformation and transmission. The Data Quality module speeds up and enhances data management by reducing the workload of time-consuming and delicate tasks. Supporting semantic data integration is also another significant contribution of this work. The Ontology Service allows users to add meaning to raw data and monitor the evolution of ontologies through versioning, which is essential to promote the quality and availability of research data over time.

The REDbox framework is constantly evolving to meet the target audience's needs, taking into account the dynamism and multidisciplinarity of the health research area. As future work progresses and as the software matures, specific comments from key users will be collected to guide the evolution of each module. Although the TB scenario motivated the solution, it applies to other health fields as well.

## Data Availability

The datasets used and/or analysed during the current study available from the corresponding author on reasonable request.
